# A comprehensive review of the articles published in the field of health in emergencies and disasters in Iran

**DOI:** 10.11604/pamj.2022.41.123.31807

**Published:** 2022-02-11

**Authors:** Hamid Reza Khankeh, Yadollah Abolfathi Momtaz, Mohammad Saatchi, Amin Rahmatali Khazaee, Abbas Naboureh, Morteza Mortazavi, Shokoufeh Ahmadi

**Affiliations:** 1 Health in Emergency and Disaster Research center, University of Social Welfare and Rehabilitation Sciences, Tehran, Iran,; 2 Department of Clinical Science and Education, Karolinska Institute, Stockholm, Sweden,; 3 Iranian Research Center on Aging, University of Social Welfare and Rehabilitation Sciences, Tehran, Iran,; 4 Malaysian Research Institute on Ageing (My Ageing), Universiti Putra Malaysia, 43400, UPM Serdang, Selangor Darul Ehsan, Malaysia,; 5 Boostan School of Nursing, Ahvaz Jundishapur University of Medical Sciences, Ahvaz, Iran

**Keywords:** Emergencies, disasters, risk management, Iran

## Abstract

**Introduction:**

Iran is one of the most disaster-prone countries in the world. A research-based approach is essential to reduce the effects of disasters and provide effective responses. This study aims to review the articles published in the field of emergencies and disasters in Iran.

**Methods:**

a combination of descriptive and qualitative content analysis using Hsieh and Shannon´s method was done. Since the first and most well-known specialized journal in the field of emergencies and disasters in Iran is the Health in emergencies and Disasters Quarterly (HDQ), all articles published in this journal were examined in terms of theme and scientometric indicators.

**Results:**

regarding the type of research, 103 were quantitative (66.5%), 18 were qualitative (11.6%), and 4 (2.6%) were performed by mixed method. Most of the articles (n=116, 76.3%) were original research. The most frequently studied risk was traffic accidents (n=17, 10.96%) followed by earthquakes (n=10, 6.45%) and floods (n=8, 5.16%). In terms of theme and content, 103 published articles were related to one of the 4 main phases of the disaster risk management cycle where most of them were related to preparedness (n=48, 46.6%) followed by mitigation (n=26, 25.24%), response (n=20, 19.42%), and recovery (n=9, 8.47%) phases.

**Conclusion:**

although there are studies related to the four phases of disaster risk management cycle in Iran, most of them are related to assessing preparedness followed by mitigation. In addition, qualitative and mixed studies could have more significant contribution to this field of research, accelerating this process requires the development of disaster research methodology training and researcher training programs as well as their organized and financial support.

## Introduction

Statistics have shown that climate change, manipulation of the nature and the rapid growth of technology have increased people's vulnerability and the rate of emergencies. Preliminary data from the Centre for Research on the Epidemiology of Disasters showed that in 2020, there were 389 natural disasters worldwide, resulting in the deaths of 15080 people, affecting 98.4 million people and causing more than $171 billion economic loss [1]. Iran is one of the most prone countries in the world for emergencies and disasters. About 93% of the regions of Iran are exposed to earthquakes and, although Iran is a dry country, 50% of its regions are exposed to floods [2]. A research-based approach is essential to reduce the effects of disasters and provide effective responses. Evidence provides the basis for managers' actions and decisions in future disasters [3]. Policymakers and governments need high-quality, evidence-based scientific data for decision-making, planning, and intervention [4]. It is only through research that we can achieve a credible scientific basis for developing mitigation programs or having proper disaster response and recovery [5]. Utilizing evidence from disasters can lead to risk reduction, greater preparedness, better response, and improved recovery and sustainable development with a return to better conditions at the local, national, and international levels. In this way, it saves human lives and reduces the damage to their property. Without systematic research, basic knowledge will not be produced and there will be no progress and development [6,7]. Moreover, considering the non-applicability of study results conducted in other countries due to cultural and social differences, accurate research-based information that takes into account the managerial and local characteristics of Iran is needed to enable policymakers and governmental agencies responsible to help minimize the effects of disasters [8]. This study aimed to provide an opportunity to better understand the issues and to identify important research gaps by reviewing the published articles in the field of emergencies and disasters in Iran. The results can help researchers and policymakers identify local capacities, disaster challenges, and opportunities, and use their knowledge and resources more appropriately to improve policies and measures for disaster prevention, risk reduction, resilience, response, and recovery.

## Methods

**Study design:** this study was done using a descriptive and qualitative content analysis based on a directed approach. The content analysis was done using Hsieh and Shannon´s method.

**Study setting and population:** since the first and most well-known specialized journal in the field of emergencies and disasters in Iran is the Health in emergencies and Disasters Quarterly (HDQ), all articles published in this journal were reviewed, furthermore, many experts in this field submit their articles to this journal and this journal is the reference of many decisions in this field. At the time of preparing this manuscript, all available articles are 155 Articles.

**Variables:** variables included type of research (qualitative or quantitative); document type (e.g. review, original, letters, subject area, case report and short communication); the time interval between articles submission, acceptance and publication, the gender of authors, study distribution of articles, keywords, scientific collaboration, research themes, and number of citations to the articles in Scopus and Google Scholar, and affiliation. Since most of the articles in the field of disaster risk management are based on four main elements including preparedness, mitigation, response and recovery, these 4 elements were considered as research themes in qualitative content analysis. Preparedness refers to activities and actions taken in advance to ensure an effective response to the impact of hazards. Mitigation refers to structural and non-structural measures to minimize the adverse effects of hazards. Response refers to the provision of assistance and interventions during or immediately after a disaster to save lives and meet the basic needs of the affected people. Finally, recovery included post-disaster measures to restore or improve the pre-disaster living conditions of the affected community [9, 10].

### Data resource and measurement

**Data collection:** descriptive variables such as type of research (qualitative or quantitative); document type (e.g. review, original, letters, subject area, case report and short communication); the time interval between articles submission, acceptance and publication, the gender of authors, study distribution of articles, keywords, scientific collaboration, and number of citations to the articles in Scopus and Google Scholar, and affiliation were collected. In the qualitative content analysis, after studying the content of the selected articles, they were coded and those related to the first 4 themes were replaced in their own themes. The codes that did not relate to the predefined themes were added into a new theme based on their similarity.

**Data analysis:** statistics analysis was performed using SPSS. In this study researchers used the descriptive statistics such as frequency and percentage for type of study, document type, scientific collaboration and research theme variables. Also, for other variables we calculate mean and standard deviation. The content analysis was done using Hsieh and Shannon´s method, considering the method suggested by Hsieh and Shannon (2005) suggested an eight-step method as follows: (1) preparation of data (2) selection of the unit or theme of analysis, (3) developing themes and the coding scheme, (4) pre-testing the coding scheme in a text sample, (5) coding all the text, (6) assessment the consistency of coding employed, (7) drawing inferences from the themes, and (8) presentation of the methods and findings [11].

**Ethical considerations:** this study was a review of articles published on an online journal. This research was not assessed by an institutional review board because this type of study does not include human subject´s research.

## Results

**Figure 1:**
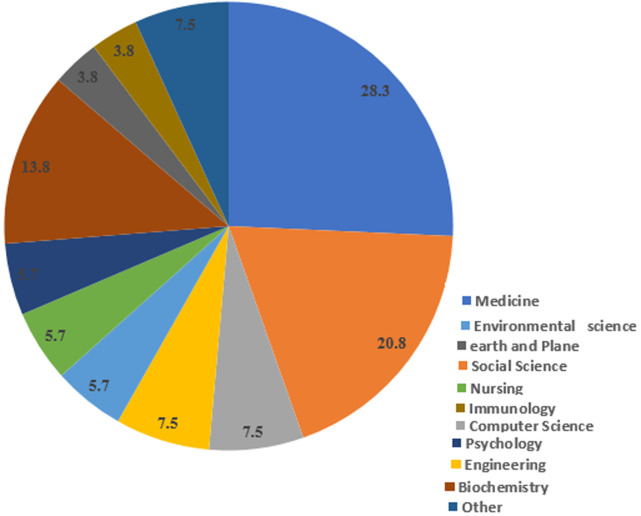
documents by subject area in HDQ in 2015-2020

**Descriptive analysis:** a total of 155 articles published from October 2015 to February 2020 in HDQ were reviewed. During this period, the mean interval between their submission and acceptance was 102±61 days and the mean interval between acceptance and publication was 58±55 days. The highest and lowest number of articles in HDQ published in 2017 (n=35) and 2015 (n=8), respectively. The words “disaster” (10%) and “accident” (5%) were the most common keywords, followed by climate change, earthquake, traffic accident, natural disaster, and preparedness. Among the provinces where the studies were conducted, Tehran with 22 articles and Kerman with 10 articles had the largest contribution. Table 1 shows information about published articles in HDQ. The average number of citations to articles was 2.5 citations per article Google Scholar. The number of citations to articles in Scopus was higher in 2020 (13 citations). Regarding the distribution of articles based on affiliation, the highest citations were for those from Iranian universities followed by the universities in Sweden. The highest number of cited articles was in the field of medicine (28.3%) followed by social sciences (20.8%) (Figure 1). Regarding the type of research, 103 were quantitative (66.5%), 18 were qualitative (11.6%), and 4 (2.6%) were performed by mixed method. In terms of the document type, most of articles were original research (n=116, 76.3%), while 15 (9.9%) were review studies, 12 (7.9%) were case studies, and 9 (5.9%) were letters to the editor. In terms of research institutions, most of the articles were written at national level with cooperation between domestic institutions and universities (n=91, 58.7%), while 49 were written with inter-university cooperation (31.6%) and 15 (9.7%) were prepared with international cooperation. The most frequently studied risk was traffic emergencies (n=17, 10.96%) followed by earthquakes (n=10, 6.45%) and floods (n=8, 5.16%).

**Table 1:** descriptive information of published articles in HDQ in 2015-2020

Variable		Frequency	Percent
**Type of study**	Quantitative	103	66.5
	Qualitative	18	11.6
	Mixed	4	2.6
	Other	30	19.419.3
	Total	155	100.0
**Document type**	Review	15	9.9
	Original	116	76.3
	Letter	9	5.9
	Case study	12	7.9
	Total	152	100.0
**Scientific collaboration**	Intra-university	49	31.6
	National	91	58.7
	International	15	9.7
	Total	155	100.0
**Theme**	Mitigation	26	25.24
	Preparedness	48	46.6
	Response	20	19.42
	Recovery	9	8.74
	Total	103	100.0

**Qualitative analysis:** in terms of theme and content, 103 published articles were related to one of the 4 main elements of the disaster risk management cycle. Most articles were related to the preparedness stage (n=48, 46.6%) followed by the mitigation stage (n=26, 25.24%), the response stage (n=20, 19.42%), and the recovery stage (n=9, 8.47%). Topics that the published articles had focused on in the field of preparedness included: awareness of disaster preparedness, assessing the impact of training on the preparedness level and preparedness programs; rapid alert system, and capacity increase, the most frequent topic in the articles was specialized training for health managers (n=7, 6.79%) [12-18]. Topics that the published articles had focused on in the field of mitigation included: risk assessment and the most frequent topic in this field was risk reduction (n=9, 8.73%) [19-27].

Topics that the published articles had focused on in the field of response were related to general health measures (n=7, 6.79%) as the most frequent topic in these articles, also triage, health of environment, water and food, psychosocial health, and management of mass casualties [28-34]. Finally, the topics that the published articles had focused on in the field of recovery included: recovery for sustainable development and attracting public participation to recover the affected community and the most frequent topic in the recovery was Physical and psychological recovery (n=5, 4.85%) [35-39]. Other articles were related to study of the occupational conditions of pre-hospital staff and factors affecting their performance, factors related to the motivation of people in disasters, disaster databases, reproductive health in disasters, air pollution, and prevention and treatment issues about COVID-19.

## Discussion

One of the indicators of the development of health research in disasters and emergencies is the number and quality of articles published in this field. This study was conducted with the aim of analyzing studies in the field of disasters and providing a general view of the evolution of research and identifying gaps and strengths in this field in Iran. Despite the fact that disasters have always occurred in nature since the beginning of life, formal scientific and academic research on disasters has begun since 1917, and epidemiological methods have been used in the study of disasters since 1970 (6). Following catastrophic emergencies and disasters such as Hurricane Katrina, September 11 attacks in the USA, the 2004 tsunami in Japan, and the Iraq war, there has been an opportunity to research and generate knowledge in this area [40], In Iran, it is not clear exactly when the official and academic scientific studies on disasters began, but it seems that it is in its early stages and research gaps are evident in many areas. One of the reasons for this can be the existence of many problems in the field of disaster research, health effects and the provision of health services. In fact, research in this field is different from conventional research [40]. Some of the most important research problems in the field of disasters are: loss of community infrastructure and difficulty accessing regions, loss of data that can be collected in the first hours after a disaster, turmoil of people and service delivery providers, risk of harm to researchers, unpreparedness or refusal of people to participate in the study, increased migration to the region and change of the target population, multifaceted nature of disasters, difference in statistical methods and study designs in disasters, researchers´ not familiarity with the methods, the conditions of affected communities [41], ethical challenges, and obtaining consent [42]. One of the ways to improve the quality of disaster studies is their support by traditional health settings such as hospitals and emergency departments. Moreover, given that the basic information of individuals are recorded before the occurrence of the disaster in these settings, it is possible to compare them with the post-disaster data [43].

Our results showed the high number of quantitative articles that are explanatory studies compared to qualitative articles that are exploratory studies. While, considering that there is little knowledge in the field of disasters, qualitative studies could have more significant contribution to this field of research. In the disaster area, qualitative researcher has to enter the field and start collecting data very quickly, because the opportunity to collect data in this setting is very limited and information may be quickly lost by, for example, internal and external migration, not remembering information and etc. (Window of opportunity) [42]. Results showed that few articles had used mixed method, while mixed method has been recommended in some studies to improve the quality of study in the field of disaster. By integrating methods and theoretical frameworks, it helps better understand the related issues [44]. The most frequently studied risk was traffic accidents followed by earthquakes and floods. Considering that traffic emergencies, earthquakes and floods are among the most common emergencies in Iran with the highest number of deaths and injuries, attention to them in articles is reasonable. Moreover, Iran is one of the countries with dangerous road driving and ranks fifth in mortality rate caused by road traffic accidents [45].

Disaster research is incomplete without studying the four phases of the disaster risk management cycle (preparedness, mitigation, response and recovery), and all phases need to be considered. In the present study, results showed that, although there were studies related to these four phases, most of the published articles were related to assessing preparedness followed by mitigation. Iran has gained considerable ability and experiences to respond to emergencies. In terms of disaster response, Iran has strong institutional and technical arrangements for disaster management at the national level. The Fifth National Development Plan (2011-2015) emphasizes the issue of disaster risk management and reduction in several areas, including increasing and improving disaster response. Disaster preparedness and mitigation are areas that require more extensive study, as well as planning by multiple sectors. Therefore, it seems that the high number of articles in these areas is completely in accordance with the needs and based on programs of national preparedness and hospital preparedness, which has been followed with great emphasis by the Ministry of Health. One of the limitations of the current study was the lack of reviewing related articles published in other journals in the field of emergencies and disasters in Iran, which caused to miss other articles published elsewhere.

## Conclusion

According to the results of this study, in Iran, it is not clear exactly when the official and academic scientific studies on disasters began, but it seems research gaps are evident in many areas. In terms of theme and content, although articles were published in all four phases of the disaster risk management cycle (preparedness, mitigation, response and recovery), most of the published articles were related to assessing preparedness followed by mitigation. In addition most studies have used quantitative methods, while, considering that there is little knowledge in the field of disasters, qualitative and mixed studies could have more significant contribution to this field of research, also the most frequently studied risk was traffic accidents followed by earthquakes and floods. Although studies in the field of disasters in Iran are growing, they are in their early stages. In order to manage disaster risk and provide effective response, it is necessary to develop disaster research methodology training, design programs for researcher training in this field, and provide organized and financial support for studies in accordance with the cultural and social conditions of Iran so that these studies can provide the basis for the actions and decisions of managers in future disasters. There are lack of disaster studies in terms of content or theme and returning to better conditions (building back better) with a special focus on sustainable development, as well as studies on philosophical issues with the aim of examining theories in the form of letters to the editor or other research types.

### What is known about this topic

- *A research-based approach is essential to reduce the effects of disasters and provide effective responses. Evidence provides the basis for managers' actions and decisions in future disasters;*
- *Disaster research is incomplete without studying the four phases of the disaster risk management cycle (preparedness, mitigation, response and recovery), and all phases need to be considered*.

### What this study adds

- *Disaster research in Iran is in its early stages and research gaps are evident in many areas;*
- *Qualitative studies can have more significant contribution to this field of disaster research in Iran since there is little knowledge in the field of disasters;*
- *Most of the published articles in Iran were related to assessing preparedness followed by mitigation*.
